# Combination of Sodium Bicarbonate (SBC) with Bacterial Antagonists for the Control of Brown Rot Disease of Fruit

**DOI:** 10.3390/jof8060636

**Published:** 2022-06-16

**Authors:** Nadia Lyousfi, Chaimaa Letrib, Ikram Legrifi, Abdelali Blenzar, Assia El Khetabi, Hajar El Hamss, Zineb Belabess, Essaid Ait Barka, Rachid Lahlali

**Affiliations:** 1 Phytopathology Unit, Department of Plant Protection, Ecole National d’Agriculture de Meknès, Km10, Rte Haj Kaddour, BP S/40, Meknès 50001, Morocco; nadia.lyousfi@usmba.ac.ma (N.L.); cletrib@enameknes.ac.ma (C.L.); ikramlegr@gmail.com (I.L.); elkhetabiassia@gmail.com (A.E.K.); hajar.elhamss@gmail.com (H.E.H.); 2 Laboratory of Plant Protection and Environment, Faculty of Sciences, Moulay Ismail University, Zitoune, Meknès 11201, Morocco; ablenzar@yahoo.fr; 3 Plant Protection Laboratory, Regional Center of Agricultural Research of Oujda, National Institute of Agricultural Research, Avenue Mohamed VI, BP428, Oujda 60000, Morocco; zineb.belabess@inra.ma; 4 Unité de Recherche Résistance Induite et Bio-Protection des Plantes-EA 4707, Université de Reims Cha-pagne-Ardenne, 51100 Reims, France

**Keywords:** *Monilinia fructigena*, antagonist bacteria, nectarine, sodium bicarbonate, combination, post-harvest fruit quality

## Abstract

Simultaneous treatment with antagonistic bacteria *Bacillus amylolquefaciens* (SF14), *Alcaligenes faecalis* (ACBC1), and the food additive sodium bicarbonate (SBC) to control post-harvest brown rot disease caused by *Monilinia fructigena*, and their effect on the post-harvest quality of nectarines were evaluated. Four concentrations of SBC (0.5, 2, 3.5, and 5%) were tested. Results showed that bacterial antagonists displayed remarkable compatibility with different concentrations of SBC and that their viability was not affected. The results obtained in vitro and in vivo bioassays showed a strong inhibitory effect of all treatments. The combination of each bacterial antagonist with SBC revealed a significant improvement in their biocontrol efficacies. The inhibition rates of mycelial growth ranged from 60.97 to 100%. These results also indicated that bacterial antagonists (SF14 or ACBC1) used at 1 × 10^8^ CFU/ mL in combination with 2, 3.5, or 5% SBC significantly improved the control of *M. fructigina* by inhibiting the germination of spores. Interestingly, disease incidence and lesion diameter in fruits treated with SF14, ACBC1 alone, or in combination with SBC were significantly lower than those in the untreated fruits. In vivo results showed a significant reduction in disease severity ranging from 9.27 to 64.83% compared to the untreated control, while maintaining the appearance, firmness, total soluble solids (TSS), and titratable acidity (TA) of fruits. These results suggested that the improved disease control by the two antagonistic bacteria was more likely due to the additional inhibitory effects of SBC on the mycelial growth and spore germination of the pathogenic fungus. Overall, the combination of both bacteria with SBC provided better control of brown rot disease. Therefore, a mixture of different management strategies can effectively control brown rot decay on fruits.

## 1. Introduction

Chemical fungicides are often used as the main method for controlling fruit diseases. However, various restrictions have been imposed over their use due to their numerous and significant side effects on human health and the environment [1,2]. The effectiveness of several fungicides such as benzimidazole and dicarboximide that are still available on the market is subsiding due to the appearance of resistant fungal strains [3,4,5,6]. In most cases, there are still no postharvest fungicides registered for some fruits despite the magnitude of losses caused by some fungal pathogens [7]. Therefore, alternative strategies for controlling postharvest pathogens of fruits are needed. During the last decades, various conventional and alternative methods have been investigated to maintain the quality of apple fruits while controlling postharvest infections [8]. These methods showed promising results, but none alone were effective as fungicides. Some antagonistic yeasts have been reported to effectively inhibit postharvest decay of fruits and vegetables and thus showed great potential as an alternative control strategy [9]. A screening of several bacteria and yeasts showed the effectiveness of several strains against postharvest pathogens (*Alternaria alternata*, *Penicillium expansum*, and *Botrytis cinerea*) and even against soil-borne diseases (*Verticillium dahliae* and *Fusarium oxysporum*) [10]. Other lactic acid bacteria were used as safe and effective biocontrol agents against *Penicillium digitatum* [11,12]. Recently, many combined treatments that can suppress post-harvest *Colletotrichum* diseases using bacteria and yeast have been tested on different fruits. The use of combined treatments is a promising alternative as antagonistic yeasts alone did not provide commercially acceptable control of fruit decay. The use of antagonists often needs to be combined with low doses of synthetic fungicides to obtain similar results to synthetic fungicides [13]. It is, therefore, necessary to devise an integrated strategy that combines several of these alternatives.

Fresh nectarines are often subjected to a rapid deterioration during pre- and post-harvest, especially when storage conditions are not well respected. Most of these losses are caused by spoilage fungi. Amongst them, brown rot–caused by *Monilinia* spp.–causes considerable economic losses in the field and during storage [14,15]. In general, post-harvest fungal diseases limit the storage period and fruit marketing. They significantly deteriorate fruit and vegetable quality, but they can be effectively controlled by synthetic chemical fungicides. However, repeated use of fungicides leads to the development of resistance by pathogens [16]. The strict requirements of sustainable agriculture, integrated crop management, and organic production made it a priority to develop alternative control methods. Restrictions have been imposed to reduce fungicide residues in fruits and vegetables and protect consumers who have become more demanding for natural organic products [17,18]. For this reason, global programs have focused on isolating effective biological control agents (BCAs) against several post-harvest fungal pathogens in many food categories. Many bacteria have also shown significant biocontrol potential, including the group *Bacillus* spp. against citrus [19], apple [20], and mongo diseases [21]. These bacteria successfully controlled the grey mold of other fruits [22]. Other strains such as *Bacillus subtilis* KLBC BS6 induced resistance to blueberry fruit to protect them against *B. cinerea* [23]. *Pseudomonas* sp. strain Y8 could significantly suppress the growth of *Ralstonia solanacearum* [24]. Yeasts also successfully controlled fungal diseases. For example, Oztekin and Karbancioglu-Guler [25] found that *Metschnikowia* yeasts controlled fungal diseases of oranges. This control was mainly through iron depletion, biofilm formation, and secretion of cell-wall degrading enzymes with volatiles. Those pathways were recognized as the major biocontrol mechanisms. However, these alternative controls when used individually have not yet achieved an acceptable level of brown rot reduction. Some of them have a poor effect against post-treatment or established infections [26].

Organic and inorganic salts are antimicrobial agents that are active against a range of phytopathogenic fungi. In particular, postharvest treatments with sodium bicarbonate (SBC, NaHCO_3_) have been proposed as a safe and effective alternative method to control postharvest rots. This salt is readily available, inexpensive, and poses little risk of phytotoxicity at concentrations ranging between 1–4% [7]. SBC has been generally reported as safe (GRAS) by the United States Food and Drug Administration and it is widely used in the food industry to enhance BCAs efficiency. Prior work has shown that the addition of these salts can improve the activity of microbial antagonists against postharvest decay of a variety of fruits [27]. Microbial antagonists have a poor ability to eradicate pre-existing infections, while SBC controlled established infections within 24 hours. SBC, however, does not exhibit persistent protection of the fruit from re-infection. The application of microbial antagonists, with or after SBC treatment, protected surface wounds from re-infections [26]. For these reasons, in this study, SBC was tested alone and in combination with antagonists to develop an efficient integrated strategy for controlling brown rot disease.

This study determined the effect of different doses of SBC on the bacterial growth of the two bacterial antagonists and then investigated the inhibitory effect of the two bacteria *Alcaligenes faecalis* (ACBC1) and *Bacillus amyloliquefaciens* (SF14) by the addition of SBC on the growth of *M. fructigena* and the germination of spores. We also investigated the effect of the treatments of antagonistic bacteria alone and in combination with SBC on the quality parameters (rot severity, weight loss, total soluble solids (TSS), titratable acidity (TA), and Maturity index) of nectarine fruits after storage in controlled conditions. These bacteria have been isolated from the soil and flowers of apple trees in Morocco. They have been identified for their antagonistic potential against *Erwinia amylovora* [28], *M. fructigena* on postharvest apples [29], and in combination with salicylic acid [30]. The combined effect of these antagonistic bacteria with SBC on postharvest diseases of fruit is however still unknown and not fully investigated.

## 2. Materials and Methods

### 2.1. Preparation of Pathogen Inoculum and Antagonists Suspensions

*M. fructigena* (VPBG) was originally isolated from sweet cherry showing typical brown rot symptoms in Serbia in 2010 [29,30]. The pathogen was then stored at 4 °C in PDA (Potato Dextrose Agar) media plates, containing 200 mL extract of boiled potatoes, 20 g dextrose, and 20 g agar in 1000 mL distilled water. For long-term storage, this fungus was maintained in 25% glycerol at −80 °C. *M. fructigena* was then freshly cultured on PDA for 7 to 10 days at 25 °C in darkness. Spore suspensions were prepared by removing the spores from a 10-day-old culture in which 3 mL of sterile distilled water (SDW) containing Tween 20 (0.05%) was added. The fungal suspension was then gently scraped on the surface of the colony with a fine scalpel to separate the mycelium spores from the PDA medium. Subsequently, the yield was filtered with sterilized Whatman paper to remove mycelial debris and to recover the spores only. The final concentration of conidial suspension was adjusted at 1 × 10^4^ spores/mL under an optical microscope (Ceti Microscopes NLCD-307B, Chalgrove, UK) with a Malassez cell (Roche, Meylan, France).

The two antagonists bacteria used in this study, *A. faecalis* ACBC1 and *B. amylolquefaciens* SF14, were originally and respectively isolated from the soil and flowers of apple trees in Morocco [28]. These bacteria were chosen due to their higher displayed efficacies against *M. fructigena* on apple fruit during post-harvest storage [29]. Both antagonists were stored at the Phytopathology Unit (ENA-Meknes) in liquid Luria-Bertani (LB) medium amended with 20% glycerol in Eppendorf tubes at −20 °C until further use. Before the experiment, each antagonist was recovered, sub-cultured on LB medium, and incubated at 28 °C in the darkness. The bacterial suspension of each bacterium was prepared from a 24-h old culture grown on an LB medium. For each bacterium, a Petri dish containing the culture of bacterial colonies in streaks was flooded with 10 mL of SDW, scraped off gently with a sterile dropper, recovered in a falcon tube (15 mL), and homogenized by vortexing. The final concentration of each bacterial antagonist was adjusted to ~2 OD (1 × 10^8^ CFU/mL) using a spectrophotometer at 420 nm (Bausch & Lomb Incorporated, Rochester, NY, USA).

### 2.2. Sodium Bicarbonate and Chemical Fungicide Preparation

SBC (NaHCO_3_) solutions were tested alone and in combination with each bacterial antagonist at different concentrations of 0.5, 2, 3.5, and 5% (*w*/*v*). The pH of these prepared concentration varies from 7.2 to 8.52. In the control (water only), the SBC concentration was at 0%.

In this study, the fungicide methyl-thiophanate (MT, 500 g/L) was used as a positive control for the in vivo experiments and was applied to the wounded fruit at a concentration of 1 ppm.

### 2.3. Fruit Preparation

Nectarine (*Prunus persica* var. *Zincal*) fruits used in these trials were hand-harvested from a commercial orchard in the area of Sefrou City, Morocco, and then transported within 5 h to the laboratory. They were picked at the harvested maturity stage and had not received any prior postharvest treatment. Before their use in the different in vivo experiments, fruits without wounds or rot were selected based on uniformity of size and absence of physical injury or disease infection. All fruit surfaces were disinfected with 2% (*v*/*v*) sodium hypochlorite for 3 min, then rinsed twice with SDW and air-dried for 1 h under a laminar flow cabinet [30].

### 2.4. Effect of NaHCO_3_ on Growth of Bacterial Antagonists

To determine the effect of different concentrations of SBC on the growth of the two antagonists, the protocol proposed by Obagwu and Korsten [31] was adopted with slight modifications. Briefly, a total of 0.7 µL of the 24h-old bacterial suspension was plated on a PDA medium containing SBC at different concentrations (0.5, 2, 3.5, and 5% *w*/*v*). The bacterial suspension was then incubated at 25 °C under shaking for 12, 24, and 48 hours to reveal the presence or absence of bacteria and to determine the effect of the treatments on the bacterial growth. Each treatment was repeated three times, and the experiment was repeated twice.

### 2.5. In Vitro Effects of NaHCO_3_, Antagonists and Their Combined Treatments on Fungal Mycelial Growth

The different concentrations of SBC were prepared by serial dilution in SDW, and then added to the PDA medium. The culture of the two antagonistic bacteria was performed using a 24-hour old culture. To evaluate the effect of the bacteria on the pathogen, a circular disk (5 mm diameter) of filter paper was placed on either side of the PDA medium containing 2.5 µL of the bacterial suspension, as previously described by Liu et al. [32]. Regarding the combined effect, a 2.5 µL suspension from each bacterium was added to the sterilized filter paper and placed on a PDA medium modified with SBC at different concentrations (0.5, 2, 3.5, and 5%). For all treatments, a mycelial plug (5 mm diameter) of *M. fructigena* was placed in the center of each Petri dish (90 mm) containing PDA medium with/without SBC and with/without each bacterium. Plates containing only PDA served as controls. All Petri dishes were incubated for 10 days at 25 °C in the dark. For each treatment, five Petri dishes were used. Mycelial growth was then assessed and recorded 5 and 10 days after incubation by measuring the diameter (mm) of fungal colonies [30]. The experiment was repeated twice and the inhibition rate (IR) was calculated according to the following formula:

IR = (DC − DT)/DC × 100

With DC: Colony diameter of the pathogenic fungus in the control treatment (PDA medium without biological treatments/SBC treatments) and DT: Colony diameter of the pathogenic fungus in bacterial treatments, SBC treatments, and their combinations.

To investigate the effect of salt, bacteria, and their combination on the structure and shape of the mycelium of *M. fructigena*, Petri dishes of the different in vitro tests were observed 10 days post-incubation using a light microscope (Ceti Microscopes NLCD-307B, Medline Scientific, Chalgrove, UK) and a magnification of 40×.

### 2.6. Impact of Biological Treatments on Spore Germination of M. fructigena

To investigate the effects of the different treatments on spore germination, in vitro measurement of germinated *M. fructigena* spores were determined according to Jemric et al. [33], with slight modification. Aliquots of 1 mL of spore suspension (5 × 10^6^ spores/mL) were transferred into 2-mL Eppendorf tubes. The spores were then incubated at 25 °C for 24 h under shaking in darkness. About 100 spores of the pathogenic fungus were examined. Each treatment was replicated three times and the experiment was repeated twice. Germination rate (PIg) was calculated as follow [34]:

Pig = (Nt − Nc)/Nt) × 100

With Nt: Total number of spores Nc: Number of germinated spores germinated.

### 2.7. In Vivo Effects of NaHCO_3_, Antagonists, and Combined Treatments on Brown Rot Disease

Disinfected nectarines were all wounded twice with a six-penny nail in their equatorial zone to reach 3 mm both in diameter and depth [35], and treated with 50 µL/wound of SBC (0.5, 2, 3.5, and 5%) alone or in combination with either antagonistic bacteria ACBC1 or SF14 (1 × 10^8^ CFU/mL). After 4 h [36], treated fruits were inoculated with a conidial suspension of the pathogen (50 µl/wound) concentrated at 1× 10^4^ spores/mL. The untreated control was only inoculated with 50 µL of SDW instead of biological treatments, while nectarines treated with 50 µL of the fungicidal methyl-thiophanate (MT, 1 ppm) served as the negative control. All fruits were then placed in a growth room chamber at 22 °C for 10 days [36]. The experiment was repeated twice over time with five replicates (5 fruits, 10 wounds per repetition) for each treatment. The disease severity was assessed 5 and 10 days after fruit inoculation. The lesion diameters were recorded using a caliper and disease severity (DS) was calculated according to the following formula [34]:

DS (%) = DT/DC × 100

With DT: average diameter (mm) of treated wounds with biological treatments/SBC/Fungicidal MT and DC: average diameter (mm) of wounds in the untreated control (inoculated only with pathogenic fungus).

### 2.8. Quality Analysis of Nectarines

#### 2.8.1. Weight Loss

The weight loss of nectarines was monitored just after treatment and then after 10 days, with 2 replicates of 5 fruits per treatment. Fruits without defects or injuries were selected, then numbered and subjected to appropriate treatment as described above. For each fruit, the mass of the nectarines was measured by an MP2000-2 balance (±0.001 g) before treatment (a) and after storage (b). The weight loss was calculated as % weight loss referenced to the initial weight of the fruit [37]. The mass loss was calculated as follows:

WL = [100 × (a − b)/a]

#### 2.8.2. Total Soluble Solids

The total soluble solids (TSS) content was determined by measuring the refractive index of the fruit with a handheld digital refractometer (Pal-1, Atago, range 0–53 Brix, least count 0.2 Brix, Japan) [38]. These measures were taken after 10 days of incubation at room temperature, around 22 °C. The results were expressed as % Brix (g per 100 g fruit weight) [36].

#### 2.8.3. Titratable Acidity

The titratable acidity (TA) of the nectarine was measured after 10 days of incubation by titration of 10 mL of fruit juice diluted with 50 mL of SDW with 0.1 mM NaOH to pH 8.1 [36] and phenolphthalein was added as an indicator. The results were expressed as in grams of malic acid per liter of juice [30].

#### 2.8.4. Maturity Index

The maturity index of nectarine fruits was recorded by the ratio between TSS and TA, as previously described [36,39].

### 2.9. Statistical Analysis

All trials were repeated twice over. All in vitro and in vivo statistical analyses were performed using SPSS software (SPSS 20.0, SPSS Inc., Chicago, IL, USA). Datasets were subjected to the analysis of variance (ANOVA). When a significant effect was revealed, the least significant difference (LSD) test was employed for means separation at a significance level of *p* < 0.05.

## 3. Results

### 3.1. Effect of NaHCO_3_ on Growth of Bacterial Antagonists

**Figure 1:**
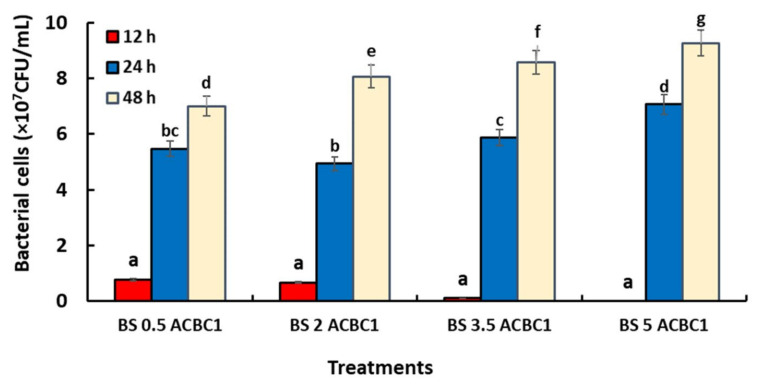
Effect of different concentrations of SBC solution (%), and duration (h) on the bacterial cell growth of ACBC1 (*Alcaligenes faecalis*) grown on PDB medium for 48 h at 25 °C under agitation. Treatments with the same letters (a–g) are not significantly different according to the LSD (*p* < 0.05) in descending order of data in Figure 1.

**Figure 2:**
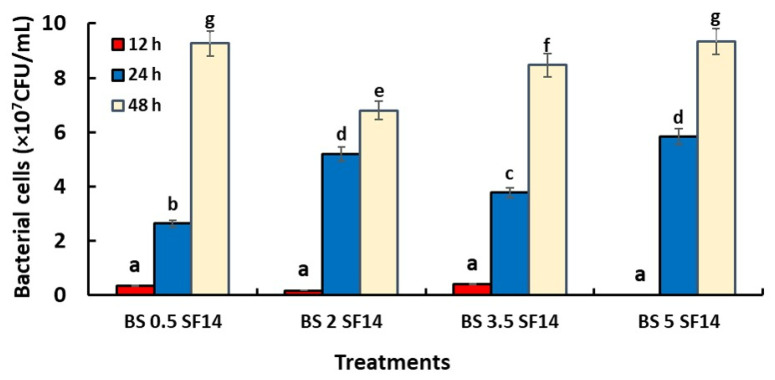
Effect of different concentrations of SBC solution (%), and duration (h) on the bacterial cell growth of SF14 (*Bacillus amyloliquefaciens*) grown on PDB medium for 48 h at 25 °C under agitation. Treatments having the same letters (a–g) are not significantly different according to the LSD (*p* < 0.05).

The effect of SBC on the population density of the two antagonistic bacteria ACBC1 and SF14 is shown in Figure 1 and Figure 2. The results obtained indicated that there was a significant difference between the treatments and all SBC concentrations on the bacterial density of ACBC1 and SF14 after 48 h of subculturing in the PDB medium. Both bacteria at the beginning of the test and during the first 12 h showed a delay in growth compared to the control. For SF14 the growth was minimal at the 2% concentration and null at a concentration reaching 5%, whilst the growth of ACBC1 was low for the 3.5% concentration and null for the 5% concentration. After 24 h, the cells of ACBC1 formed increased compared with the first hours and varied between 5.45 × 10^7^ CFU/mL for the 0.5 concentration and 6.25 × 10^7^ CFU/mL for 5% and continued to increase after 48 h. For the antagonistic bacteria SF14, after 24 hours, the number of colonies formed in the different concentrations of SBC solution varied between 3.45 × 10^7^ CFU/mL for 0.5% and 5.9 × 10^7^ CFU/mL for 5% and also continued to grow after 48 hours of incubation period until reaching 9.2 × 10^7^ CFU/mL at the 5% concentration. 

### 3.2. In Vitro Effect of Sodium Bicarbonate, Antagonistic Bacteria, and Their Combinations on Fungal Growth

The results showed that the inhibition rates of mycelial growth were dependent on the treatments and incubation period (after 5 and 10 days) (Table 1). 

The results of the two-factor analysis of variance showed a significant effect of the interaction on the mycelial growth of M. fructigena. According to Dunnett’s test, the treatments used are significantly different compared to the control. Regardless of incubation time, complete inhibition was recorded in 3.5% SBC + SF14 and 5% SBC + SF14 treatments, making them ideal candidates for prolonged protection. The majority of treatments showed different rates of inhibition according to the time of incubation. After 5 days, the highest rate was in the treatment 5% SBC reaching 96.58% but decreased after 10 days to reach 92.87%, indicating that the efficacy of the treatments is time-dependent. 

### 3.3. Effect of Treatments on the Mycelial Structure and Spore Germination of M. fructigena

**Figure 3:**
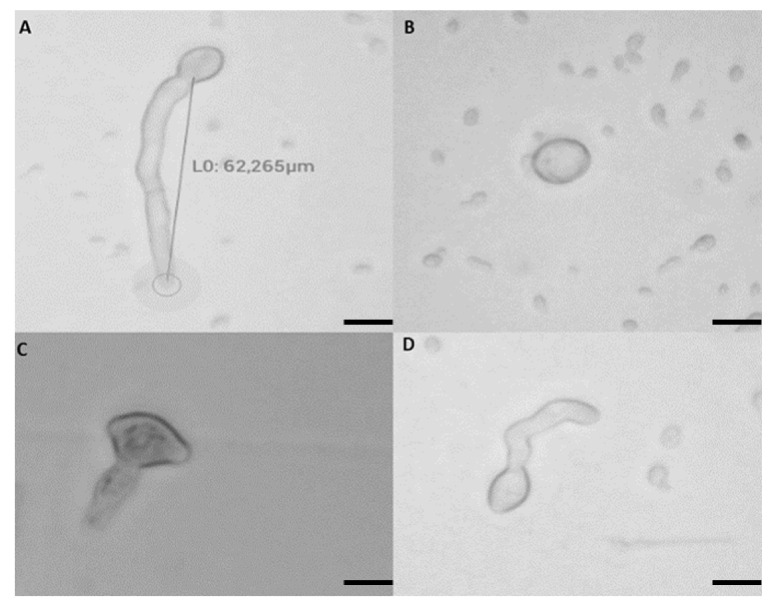
Microscopic observations of spores of *Monilinia fructigena* species after 24 h of incubation at 25 °C with agitation according to treatments. Normal and germinated spore in the untreated control (**A**). Non-germinated spores in 5% SBC + ACBC1 treatment (**B**). Altered spore with the appearance of germ tube in 3.5% SBC + SF14 treatment (**C**). Normal spore and appearance of germ tube in 5% SBC treatment (**D**). Bars = 10 µm.

Microscopic examination of the mycelium from the inhibition zones displayed substantial changes and degradation in hyphal structures, cellular lysis, hyphal swelling, and vacuolation when compared with mycelium of the pathogenic fungus from the untreated control. In addition, Figure 3 also shows the impact of biological treatments on the structure of the spores after 24 h of incubation time. The results in Table 2 indicate that the germination of *M. fructigena* spores was completely inhibited when treated with 2% SBC + ACBC1, 5% SBC + ACBC1, and chemical fungicide. While the inhibition for the treatments 3.5% SBC + SF14, 5% SBC + SF14 and 3.5% SBC + ACBC1 reached 91.86%, 99.18% and 91.05%, respectively.

### 3.4. In Vivo Effect of SBC, Bacteria, and Their Combinations on Brown Rot Disease

Table 3 illustrates the severity of brown rot recorded for each treatment during two incubation periods (5 and 10 days) and their corresponding lesions diameters. A two-way analysis of variance of the different lesion diameters per treatment showed that there was a significant effect of the interaction between treatments. A significant difference between the control and the other treatments was then confirmed by Dunnett’s test. The pathogenicity of the fungus evolved, and this was translated into a change in the severity of the disease over time. Treatments including 5% SBC, 2% SBC + ACBC1, 3.5% SBC + ACBC1, ACBC1, 3.5% SBC + SF14, and 0.5% SBC + SF14 showed a severity reaching 13.69, 12.95, 14.39, 15.09, 16.47, and 18.89%, respectively. However, the 0.5% SBC treatment showed a maximum severity reaching 64.83% compared to the control after 10 days of incubation.

### 3.5. Effect of Biological Treatments on Quality Parameters of Nectarines Fruit

#### 3.5.1. Weight Loss

There is a significant effect of treatment on weight loss according to the ANOVA test. In addition, treatments including 2% SBC + ACBC1 and 5% SBC were significantly different from the control according to the LSD test and showed a low weight loss compared with the control (0.16) (Table 4).

#### 3.5.2. Total Soluble Solids (Brix, TSS)

The one-factor analysis of variance revealed the significant effect of the treatment on the rate of soluble solids. Thus, no significant difference between the control and ACBC1, 5% SBC-SF14, 5% SBC, and 2% SBC + ACBC1 treatments was demonstrated by the LSD test. However, combinations with 2% SBC marked high TSS values compared with the control (Table 4).

#### 3.5.3. Titratable Acidity (TA)

The effect of the treatments on titratable acidity was significant according to ANOVA (Table 4). Treatments were also significantly different from the control according to the LSD test except for 3.5% SBC, ACBC1, and 2% SBC + ACBC1. The highest titratable acidity was recorded in the treatment 3.5% SBC + ACBC1 reaching 12.37 g malic acid/L juice, while the treatment 3.5% SBC + SF14 recorded a value of 8.04 g malic acid/L juice.

## 4. Discussion

Great progress has been made in the biological control of postharvest diseases using various microorganism species. Currently, the trend is towards using a combination of various approaches, which was proved to be more effective and comparable in efficacy to chemical fungicides. SBC increased the effectiveness of decay control by some antagonists alone or in combination [40]. In this study, we investigated the feasibility of a combined application of a microbial antagonist (ACBC1 and SF14) and SBC to control post-harvest brown rot caused by *M. fructigena*.

The confrontation experiment between the antagonists and the different concentrations of SBC showed that the growth of bacteria was both time- and dose-dependent. The viability of SF14 and ACBC1 was minimal in the first 12 h of incubation, then increased after 24 h and 48 h. The bacterial growth increased in the different concentrations, indicating that the two antagonists can survive in high concentrations of the SBC. Similarly, Papavasileiou et al. [27] proved that SBC, at the different incubation times, did not show any negative effect on the viability of L47 at the tested concentration reaching 1%. A similar population density was assessed for the salt and water suspensions of the antagonist [27]. Additionally, Hong et al. [35] showed that a concentration reaching 2% of SBC was efficient in controlling postharvest decay of mandarin fruits, reducing up to 80% of fruit decay compared to the control. The concentration of 2% of SBC was also compatible when combined with *B. amyloliquefaciens* HF-01, making it an ideal candidate for integrated control of postharvest decay [35]. In another study controlling anthracnose rot in loquat fruit where another compound was tested, they also found compatibility between the CaCl_2_ and the antagonistic bacteria when combined [13]. The combined treatment of both *P. membranifaciens* and a concentration reaching 2% of CaCl_2_ resulted in significantly improved control of the disease in comparison with the treatment of *P. membranifaciens* or CaCl_2_ alone [13]. A combination of CaCl_2_ with *P. membranifaciens* did not influence the population density of this antagonist even after 3 or 6 days of incubation [13].

The results of the in vitro tests showed that the treatments of both antagonistic agents alone, or of SBC when also applied alone at different doses, were able to inhibit the mycelial growth of the pathogen. The results obtained by the combined treatments, however, gave better inhibition of mycelial growth. A complete inhibition rate of mycelial growth for the treatments 3.5% SBC + SF14 and 5% SBC + ACBC1 reaching 100% were observed after 10 days of incubation. For the combinations 2% SBC + SF14, 5% SBC + SF14, 2% SBC + ACBC1, and 3.5% SBC + ACBC1, the inhibition rate was only around 90%. Therefore, these treatments are complementary to one another when applied in combination. Our results agree with the research carried out by De Costa and Gunawardhana [41], where they found that the addition of SBC reduced mycelial growth, spore production, and also spore germination of *Colletotrichum musae* under in vitro conditions. Other studies have reported the inhibitory effect of *Bacillus* spp. on the growth of a large number of phytopathogenic agents by antagonisms. Amongst them, grey rot and brown rot were also tested in combination with SBC. Similarly, the combination of SBC with *B. subtilis* effectively controlled the ring rot of stored pear and gave the greatest biocontrol effects against *B. berengeriana*. *Aureobasidium pullulans* strain L47 significantly reduced *Botrytis* rot by 98%, and by around 94% when it was combined with SBC [27]. 

In this study, the highest inhibition percentages (>90%) of combination treatments were generated by the mixture of *A. faecalis* (ACBC1) with SBC or *B. amyloliquefaciens* (SF14) with SBC at concentrations of 2, 3.5, and 5%. Similarly, in sweet cherry, Karabulut et al. [42] proved that a concentration reaching 0.03 M and 0.06 M of SBC was effective in inhibiting the growth of *B. cinerea* and *P. expansum*. Obagwu and Korsten [31] showed that the addition of 1% SBC with *B. subtilis* resulted in a significant improvement in the biocontrol activity of all isolates [7]. A concentration reaching 2% of SBC was, however, more effective in reducing the severity of blue rot in *Penicillium expansum* compared to 0.3 or 1% [7]. The combination of SBC with *Pichia membranefaciens* and *Cryptococcus laurentii* showed a significant inhibitory effect against *M. fructicola* at all concentrations (0.5, 1, 2, and 4%) [40]. A significant increase in the biocontrol activity of *Cryptococcus laurentii* (ST4-E14) and *Metschnikowia pulcherrima* (FMB-24H-2) isolates against *P. expansum* on apple fruit was observed when the isolates were combined with SBC [7]. 

In some cases, higher concentrations of SBC could significantly affect the survival of antagonists, indicating that the choice of an effective concentration is crucial in a successful biological control program. In our experience, the concentration of 2, 3.5, and 5% of SBC in combination with ACBC1 or SF14 gave excellent mycelial inhibitions without affecting the survival of both ACBC1 and SF14. These concentrations can therefore be recommended in future management programs for post-harvest disease. Papavasileiou et al. [27] suggested that the improvement of biocontrol activity when in combination with SBC could be due to its ability to tolerate high salt concentrations compared to that of fungal pathogens.

Our study also showed that the studied treatments affected the germination capacity of the spores of the pathogen. The treatments 3.5% SBC + ACBC1 and 5% SBC + ACBC1 were able to completely inhibit the germination of the pathogen spores. The inhibition of germed spores in other treatments was around 90%. Microscopic observation of the mycelium of *M. fructigena* revealed alterations in the shape of the mycelium under the effect of the two antagonists (SF14 and ACBC1) added to 3.5% SBC and 2% SBC. Deformation of the mycelium and even degradation of the mycelial wall with destruction were detected under the effect of the 3.5% SBC + ACBC1 treatment. Similarly, Dihazi et al. [43] revealed that *Bacillus* strains can parasitize phytopathogenic microorganisms by degrading their walls, including *B. amyloliquefaciens* against *Fusarium oxysporum* f. sp. *Albedinis*.

In this study, the 0.5% SBC treatment showed a maximum severity reaching 64.83% whilst the lowest was in the 2% SBC + SF14 treatment compared with the control after 10 days of incubation. Our study gave promising results in also directly controlling the fruits in controlled conditions. Mechanisms and synergistic interactions behind the combined treatment should however be investigated further. Similarly, the yeast *Hanseniaspora uvarum* in combination with SBC was effective in controlling gray mold [38]. In apple fruits, treatments with this antagonist in combination with SBC reduced the incidence of blue mold from 84% to 97%, a reduction higher than that of the antagonist alone [44]. The yeast *Metschnikowia fructicola* and SBC, in combinations, were applied to table grapes on vines 24 h before harvest to control the incidence of postharvest disease, and significantly reduced the total number of decayed berries caused by *B. cinerea*, *Alternaria* spp., or *Aspergillus niger* after storage for 30 days at 1 °C followed by 2 days at 20 °C. 

In vivo tests revealed a significant effect on nectarine weight loss compared to the control. The SBC at a concentration reaching 5% and its combination with ACBC1, reduced fruit loss. However, combinations with 2% SBC recorded high TSS values compared to the control. With the combination of SBC and SF14, and the bacteria used alone, a reduction in titratable acidity of 1.43 and 0.06, respectively, was observed. The results also showed that the combination of treatments did not greatly modify the fruit quality parameters. The maturity index showed a difference between the different treatments and the control, with the highest value recorded in the control. This index is good for evaluating fruit ripening. Similarly, the nectarine maturity index increased during storage and was higher in the control than in treated nectarines [39]. A study has shown that the use of *Kluyveromyces marxianus* bacterium in combination with 2% SBC showed no significant effect on weight loss, total soluble solids, and titratable acidity after storage for 15 days at 20 °C [36]. Similar results were confirmed by Hong et al. [35] with the combination of *B. amyloliquefaciens* HF-01 with 2% SBC. The integration of *Hanseniaspora uvarum* with SBC significantly reduced weight loss while maintaining the appearance of the fruit, total soluble solids content, and titratable acidity of the grapes at 2 ± 1 °C, HR 90–95% during the 10-day storage period [38]. The treatment comprising *B. amyloliquefaciens* combined with 2% SBC was as effective as the fungicide treatment and reduced decay to less than 80% compared to the control. *B. amyloliquefaciens* HF-01 in combination with 2% SBC significantly reduced postharvest decay without impairing fruit quality after storage at 25 °C for 4 weeks or at 6 °C for 8 weeks [35].

The combination of different alternative control methods has demonstrated a large potential in post-harvest disease control–especially the combination of yeasts/bacteria with approved chemical compounds. Similarly, in cherry fruits, a combination of both copper hydroxide and lime sulfur with *Aureobasidium pullulans* showed a reduction in the incidence of brown rot blossom blight [45]. Furthermore, a combination of LS11 strain of the yeast *Rhodotorula kratochvilovae* and *B. subtilis* strain QST 713, and a concentration of 25% of cyprodinium, cyprodinil, and boscalid sufficiently protected the treated peach with minimum chemical residues left in the peach juice [46]. Therefore, an effective combination of different biological and chemical methods requires a good understanding of the ecology of antagonists [47]. Understanding how to manage postharvest diseases cost-effectively and reliably and how different alternative technologies affects the host and the microorganism community is becoming more crucial in successful management programs.

## 5. Conclusions

In conclusion, the results highlighted the combinations that increased the inhibitory potential of the bacteria on the growth of *M. fructigena* in vitro and in vivo, as well as their effect on the quality parameters of the studied fruits (weight loss, total soluble solids, and titratable acidity, maturity index).

We found that treatment with the combination of the two antagonistic strains of SF14, ACBC1, and SBC had a significant impact on the control of brown rot in nectarine fruit. In vitro results showed that the control effect was associated with inhibition of *M. fructigena* growth and pathogen spore germination. In vivo tests showed that all treatments did not modify the quality parameters of nectarines. In general, the combination of SF14, ACBC1, and SBC was more effective in controlling brown rot affecting nectarine fruits than individual treatments. It can thus provide a reliable solution for the control of brown rot during commercial storage.

## Figures and Tables

**Table 1:** Inhibition rate of mycelial growth (%) of *M. fructigena* obtained by SBC concentrations, antagonistic bacteria (*Bacillus amylolquefaciens* SF14 and *Alcaligenes faecalis* ACBC1), and their combinations after 5 and 10 days of incubation at 25 °C in darkness.

Treatments	pH	5 Days of Incubation		10 Days of Incubation	
		Colony Diameter (mm)	IR (%)	Colony Diameter (mm)	IR (%)
Untreated Control	PDA_pH_ = 7.02	54.03 ^e^	0.00	82.75 ^f^	0.00
0.5% SBC	8.24	27.42 ^d^	54.28	34.91 ^e^	60.97
2% SBC	8.34	12.83 ^ab^	84.03	14.15 ^abcd^	88.24
3.5% SBC	8.38	7.84 ^ab^	94.20	10.65 ^ab^	92.74
5% SBC	8.52	6.44 ^a^	96.58	10.54 ^ab^	92.87
SF14	7.22	12.01 ^ab^	86.62	16.74 ^bcd^	84.91
0.5% SBC + SF14	7.11	9.98 ^ab^	79.67	19.57 ^cd^	81.26
2% SBC + SF14	7.20	9.43 ^ab^	91.05	9.81 ^ab^	93.81
3.5% SBC + SF14	7.25	5.00 ^a^	100.00	5.00 ^a^	100.0
5% SBC + SF14	7.23	6.88 ^a^	95.06	7.71 ^ab^	96.51
ACBC1	7.11	17.27 ^bc^	73.48	20.13 ^cb^	80.54
0.5% SBC + ACBC1	7.21	21.16 ^cd^	67.03	22.24 ^d^	77.83
2% SBC + ACBC1	7.22	7.34 ^ab^	93.91	10.27 ^ab^	93.23
3.5% SBC + ACBC1	7.30	10.77 ^ab^	86.94	12.58 ^abc^	90.25
5% SBC + ACBC1	7.34	5.00 ^a^	100.00	5.00 ^a^	100.0

The data are the average of two independent trials with four replicates for each pathogen-treatment combination. *Bacillus amylolquefaciens* (SF14), *Alcaligenes faecalis* (ACBC1), and Sodium bicarbonate (SBC). The mean diameters with the same letters (a–f) are not significantly according to the LSD test (*p* < 0.05) in descending order of data in Table 1.

**Table 2:** Inhibition rate of germination (%) of *M. fructigena* obtained by sodium bicarbonate (SBC), antagonistic bacteria (*Bacillus amylolquefaciens* SF 14 and *Alcaligenes faecalis* ACBC1), and their combinations, after 24 h of incubation at 25 °C in darkness with agitation.

Treatments	Inhibition Rate of Spore Germination (%)
0.5% SBC	36.17 ± 3.72 ^a^
2% SBC	60.97 ± 2.11 ^c^
3.5% SBC	56.91 ± 2.81 ^c^
5% SBC	60.56 ± 4.92 ^c^
SF14	71.54 ± 5.08 ^d^
0.5% SBC + SF14	35.77 ± 3.92 ^a^
2% SBC + SF14	86.58 ± 4.39 ^e^
3.5% SBC + SF14	91.86 ± 0.70 ^e^
5% SBC + SF14	99.18 ± 1.40 ^f^
ACBC1	73.98 ± 1.86 ^d^
0.5% SBC + ACBC1	43.90 ± 3.22 ^b^
2% SBC + ACBC1	100 ± 0.00 ^f^
3.5% SBC + ACBC1	91.05 ± 1.40 ^e^
5% SBC + ACBC1	100 ± 0.00 ^f^
Methyl-Thiophanate (1 ppm)	100 ± 0.00 ^f^

Values are the means of two trials over time with three replicates (*Bacillus amylolquefaciens* (SF14), *Alcaligenes faecalis* (ACBC1), and Sodium bicarbonate (SBC)). Inhibition rates with the same letters (a–f) are not significantly according to the LSD test (*p* < 0.05) in descending order of data in Table 2.

**Table 3:** Disease Severities (DS%) of brown rot on nectarines artificially wound-inoculated (*M. fructigena* at 1 × 10^4^ conidia/mL) obtained by sodium bicarbonate, antagonistic bacteria (*Bacillus amylolquefaciens* SF14 and *Alcaligenes faecalis* (ACBC1), and their combinations after 5 and 10 days of incubation at 22 °C.

Treatments	1 × 10^4^ Spores/mL			
	5 Days of Incubation		10 Days of Incubation	
	Lesion Diameter (mm)	DS (%)	Lesion Diameter (mm)	DS (%)
Untreated Control	58.08 ^e^	100.00	68.27 ^e^	100.00
0.5%SBC	32.91 ^d^	56.66	44.26 ^d^	64.83
2% SBC	20.14 ^c^	34.68	32.70 ^c^	47.89
3.5% SBC	5.93 ^ab^	10.21	17.34 ^b^	25.40
5% SBC	4.76 ^ab^	8.20	9.34 ^ab^	13.69
ACBC1	5.35 ^ab^	9.22	10.30 ^ab^	15.09
0.5% SBC + ACBC1	14.23 ^bc^	24.49	18.99 ^b^	27.81
2% SBC + ACBC1	5.08 ^ab^	8.75	8.84 ^ab^	12.95
3.5% SBC + ACBC1	7.64 ^ab^	13.15	9.82 ^ab^	14.39
5% SBC + ACBC1	12.09 ^abc^	20.82	18.04 ^b^	26.43
SF14	11.50 ^abc^	19.80	18.16 ^b^	26.60
0.5% SBC + SF14	9.10 ^abc^	15.67	12.90 ^b^	18.89
2% SBC + SF14	5.00 ^ab^	8.61	6.33 ^ab^	9.27
3.5% SBC + SF14	7.46 ^ab^	12.84	11.25 ^ab^	16.47
5% SBC + SF14	0.00 ^a^	8.61	0.00 ^a^	25.63
Methyl-Thiophanate (1 ppm)	0.00 ^a^	0.00	0.00 ^a^	0.00

The data are the average of two independent trials with five replicates (5 fruits, 10 wounds) for each pathogen-treatment combination. *Bacillus amylolquefaciens* (SF14), *Alcaligenes faecalis* (ACBC1), and Sodium bicarbonate (SBC). The mean diameters with the same letters (a–e) in the descending order of data in Table 3 are not significantly different according to the LSD test (*p* < 0.05).

**Table 4:** Effect of sodium bicarbonate, antagonistic bacteria (*Bacillus amylolquefaciens* SF14 and *Alcaligenes faecalis* ACBC1) on quality parameters of nectarine fruits artificially wound-inoculated (3 mm × 3 mm wounds; *M. fructigena* at 1 × 10^4^ conidia/mL) during 10 days of storage at 22 °C.

Treatments	Fruits Appearance	Damage to Pericarp (+/−)^a^	Quality Parameters			
			Weight Loss	Total Soluble Solids (%)	Titratable Acidity (g/L Malic Acid)	Maturity Index
Untreated Control	rotten fruit	+	0.127 ± 0.01 ^ab^	10.67 ± 0.289 ^cd^	9.53 ± 0.00 ^d^	1.10
0.5% SBC	brownish spots	+	0.157 ± 0.00 ^c^	7.14 ± 0.24 ^a^	11.34 ± 0.08 ^hi^	0.60
2% SBC	beginning of lesions	+	0.15 ± 0.02 ^bc^	8.81 ± 0.33 ^b^	11.41 ± 0.04 ^i^	0.81
3.5% SBC	dark orange	−	0.150 ± 0.03 ^bc^	12.77 ± 0.59 ^ef^	10.05 ± 0.00 ^e^	1.30
5% SBC	dark orange	−	0.117 ± 0.00 ^a^	10.70 ± 0.75 ^cd^	10.94 ± 0.19 ^g^	1.00
ACBC1	circular stains	+	0.137 ± 0.01 ^abc^	10.33 ± 0.58 ^c^	10.12 ± 0.13 ^e^	1.00
0.5% SBC + ACBC1	brownish spots	+	0.163 ± 0.00 ^c^	9.00 ± 0.00 ^b^	11.03 ± 0.4 ^gh^	0.81
2% SBC + ACBC1	dark orange	−	0.13 ± 0.00 ^ab^	11.00 ± 0.00 ^cd^	10.61 ± 0.33 ^f^	1.00
3.5% SBC + ACBC1	pale orange	−	0.147 ± 0.01 ^bc^	9.23 ± 0.21 ^b^	12.37 ± 0.04 ^j^	0.75
5% SBC + ACBC1	dark orange	−	0.127 ± 0.01 ^ab^	13.10 ± 0.26 ^f^	8.88 ± 0.14 ^c^	1.44
SF14	pale orange	−	0.14 ± 0.00 ^abc^	9.10 ± 0.14 ^b^	9.62 ± 0.67 ^d^	0.90
0.5% SBC + SF14	beginning of lesions	+	0.16 ± 0.00 ^c^	13.30 ± 0.00 ^f^	8.44 ± 0.15 ^b^	1.62
2% SBC + SF14	dark orange	−	0.117 ± 0.00 ^a^	13.13 ± 0.231 ^f^	8.75 ± 0.08 ^bc^	1.44
3.5% SBC + SF14	pale orange	−	0.153 ± 0.00 ^bc^	12.10 ± 0.173 ^e^	8.04 ± 0.00 ^a^	1.50
5% SBC + SF14	dark orange	−	0.160 ± 0.01 ^c^	11.30 ± 0.00 ^d^	7.93 ± 0.43 ^a^	1.37
Methyl-thiophanate-(1 ppm)	healthy fruit	−	0.16 ± 0.00 ^c^	12.73 ± 0.643 ^ef^	10.18 ± 0.10 ^e^	1.30

^a^ “+” pericarp damaged and “−” pericarp undamaged. The data are the average of two independent trials with five replicates (5 fruits, 10 wounds) for each pathogen-treatment combination. SF14: *Bacillus amylolquefaciens*, ACBC1: *Alcaligenes faecalis*, and SBC: Sodium bicarbonate. The mean diameters with the same letters are not significantly different according to the LSD test (*p* < 0.05).

## Data Availability

The data presented in this study are available on request from the corresponding author. The data are not publicly available due to restrictions, e.g., privacy or ethical issue.
